# Hymenoptera of Canada

**DOI:** 10.3897/zookeys.819.28510

**Published:** 2019-01-24

**Authors:** Andrew M.R. Bennett, Cory S. Sheffield, Jeremy R. deWaard

**Affiliations:** 1 Canadian National Collection of Insects, Arachnids and Nematodes, K.W. Neatby Bldg., 960 Carling Avenue, Ottawa, Ontario, K1A 0C6, Canada Canadian National Collection of Insects Ottawa Canada; 2 Royal Saskatchewan Museum, 2340 Albert Street, Regina, Saskatchewan, S4P 2V7, Canada Royal Saskatchewan Museum Regina Canada; 3 Centre for Biodiversity Genomics, University of Guelph, 50 Stone Road East, Guelph, Ontario, N1G 2W1, Canada University of Guelph Guelph Canada

**Keywords:** ants, bees, biodiversity assessment, Biota of Canada, DNA barcodes, Hymenoptera, survey, wasps

## Abstract

A summary of the numbers of species of the 83 families of Hymenoptera recorded in Canada is provided. In total, 8757 described species are recorded compared to approximately 6000 in 1979, which is a 46% increase. Of the families recognized in 1979, three have been newly recorded to Canada since the previous survey: Anaxyelidae (Anaxyleoidea), Liopteridae (Cynipoidea), and Mymarommatidae (Mymarommatoidea). More than 18,400 BINs of Canadian Hymenoptera are available in the Barcode of Life Data Systems (Ratnasingham and Hebert 2007) implying that nearly 9650 undescribed or unrecorded species of Hymenoptera may be present in Canada (and more than 10,300 when taking into account additional species that have not been DNA barcoded). The estimated number of unrecorded species is very similar to that of 1979 (10,637 species), but the percentage of the fauna described/recorded has increased from 36% in 1979 to approximately 45% in 2018. Summaries of the state of knowledge of the major groups of Hymenoptera are presented, including brief comments on numbers of species, biology, changes in classification since 1979, and relevant taxonomic references.

## Introduction

Hymenoptera constitutes one of the most speciose orders in Canada and the world (Forbes et al. 2018). During the last assessment of this order in Canada (Masner et al. 1979), 6028 species were reported, although the approximately 80 species in the family Eurytomidae were inadvertently omitted and the numbers of species of Platygastroidea, Ceraphronoidea, Bethylidae, Cynipoidea and Pompilidae were overestimated as it appears that undescribed species were included. Thus, the known richness in 1979 was approximately 6000 species. The most comprehensive faunal inventory of Hymenoptera in Canada is the *Catalog of the Hymenoptera in America North of Mexico* (Krombein et al. 1979) which listed general distributions of species up to 1972 to 1976 (the cut-off date depending on the superfamily). No complete distributional survey of the species of Hymenoptera in North America (or Canada) has followed, although the species lists and distributions on which the analysis in this manuscript is based will be published in a forthcoming series of checklists of the Hymenoptera of Canada, Alaska, and Greenland (A Bennett unpubl. data). Nonetheless, a tremendous amount of data has been produced since 1979 on the taxonomy, nomenclature, and distribution of particular groups of Hymenoptera, including the presence of species in Canada. Some of the most important sources are noted in the respective sections on major taxa (see below as well as in Table 1). In some instances in which major references were not included in Masner et al. (1979), these references have also been included in Table 1.

**Table 1.** Census of Hymenoptera in Canada.

Taxon^1^	No. species reported in Masner et al. (1979)	No. species currently known from Canada	No. BINs^2^ available for Canadian species	Est. no. undescribed or unrecorded species in Canada^3^	General distribution by ecozone^3A^	Information sources^4^
**Sawflies (previously suborder Symphyta)**						Smith 1979a, Goulet 1987, Taeger et al. 2010, Blank et al. 2012
**Superfamily Anaxyleoidea**						
Anaxyelidae	0	1	0	0	Western Interior Basin	Goulet 1992
**Superfamily Cephoidea**						
Cephidae	16	12	8	0	all except Arctic	Ries 1937, Smith 1986, Smith and Solomon 1989, Smith and Schiff 2005
**Superfamily Orussoidea^5^**						
Orussidae	3	5	4	0	south of Boreal Cordillera and Taiga ecozones, except Atlantic Maritime	Middlekauff 1983, Skvarla et al. 2015
**Superfamily Pamphilioidea^6^**						
Pamphiliidae	47	54	33	0	all except Arctic	Middlekauff 1958, 1964, Eidt 1969
**Superfamily Siricoidea^5^**						
Siricidae	14	20	25	4	all except Arctic	Schiff et al. 2012, Goulet et al. 2015
Xiphydriidae	5	7	3	0	all except Arctic	Smith 1976
**Superfamily Tenthredinoidea**						
Argidae	14	27	17	0	all except Arctic	Smith 1969a, 1971a, 1989
Cimbicidae	4	8	15	7	southern Arctic and south	Smith 1979a
Diprionidae	22	25	14	0	southern Arctic and south	Ross 1955, Smith 1974
Pergidae	5	3	4	1	Mixedwood Plains, Atlantic Maritime	Smith 2006
Tenthredinidae	300	532	528	200*	all ecozones	Goulet 1986, 1996, Smith 1969b, c, 1971b, 1979b
**Superfamily Xyeloidea^6^**						
Xyelidae	13	16	11	0	all except Arctic	Burdick 1961, Smith 1977, Smith and Schiff 1998
**Total Sawflies**	**443**	**710**	**662**	212*		
**Suborder Apocrita**						
**Superfamily Ichneumonoidea**						
Braconidae	830	1165	3411	2246	all ecozones	Marsh 1979a, b, Wharton et al. 1997, Yu et al. 2016
Ichneumonidae	2001	3037	4748	1705*	all ecozones	Carlson 1979c, Dasch 1979, 1984, 1988, 1992, Townes 1983, Townes et al. 1992, Schwarzfeld 2014, Yu et al. 2016
**Total Ichneumonoidea**	**2831**	**4202**	**8159**	3951*		
**Superfamily Diaprioidea^7^**						
Diapriidae ^8^	150	177	760	583	southern Arctic and south	Masner 1991, 1993a, Johnson 1992, Masner and García 2002, Sharkey 2007, Sharkey et al. 2012
Diapriidae ^8^	?	8	3	0	south of Arctic and Boreal Cordillera	Masner 1976a
**Total Diaprioidea**	**150**	**185**	**763**	**583**		
**Superfamily Platygastroidea^9^**						
Platygastridae ^10^	250	160	2287	2127	southern Arctic and south	Masner 1976b, 1983a, b, Townes and Townes 1981, Ritchie and Masner 1983, Johnson 1992, 2018
**Superfamily Proctotrupoidea^11^**						
Heloridae	2	2	2	0	all except Arctic	Townes 1977b, Johnson 1992
Pelecinidae	1	1	1	0	Boreal Shield, Mixedwood Plains, Atlantic Maritime	Johnson 1992, Johnson and Musetti 1999
Proctotrupidae	60	67	63	0	southern Arctic and south	Townes and Townes 1981, Johnson 1992
Roproniidae	2	2	1	0	Boreal Shield, Mixedwood Plains	Townes 1948, Johnson 1992
Vanhorniidae	1	1	0	0	Boreal Shield, Mixedwood Plains	Townes and Townes 1981, Johnson 1992
**Total Proctotrupoidea**	**66**	**73**	**67**	**0**		
**Superfamily Chalcidoidea**						Gibson et al. 1997, Noyes 2017
Aphelinidae	30	37	130	93	all ecozones	Gordh 1979, Rosen and Debach 1979, Schauff et al. 1996, Shirley et al. 2017
Azotidae ^12^	?	1	1	0	Mixedwood Plains	Jarvis 1907, Heraty et al. 2013
Chalcididae	22	37	18	0	southern Arctic and south	Burks 1979d, Halstead 1990,Delvare and Bouček 1992
Encyrtidae	65	102	265	163	all ecozones	Trjapitzin and Gordh 1978a, b, Gordh 1979, Noyes and Woolley 1994
Eucharitidae	10	7	6	0	all except Arctic	Burks 1979h, Heraty 1985, 2002
Eulophidae ^13^	173	373	1373	1000	all ecozones	Burks 1979h, Yoshimoto 1983, Peck 1985, Schauff 1991, LaSalle 1994, Triapitsyn and Headrick 1995, Hansson 1987, 1988, 1989, 1994a, b, 1995, 1996a, b
Eupelmidae	14	29	24	0	south of Taiga ecozones	Burks 1979g, Gibson 1989, 1995, 2002, 2010, 2011
Eurytomidae ^14^	0	90	107	17	southern Arctic and south	Bugbee 1967, Burks 1979c, Zhang et al. 2017
Leucospidae	1	1	1	0	Pacific Maritime, Western Interior Basin, Prairies, Boreal Shield, Mixedwood Plains, Atlantic Maritime	Bouček 1974, Burks 1979e
Megastigmidae ^15^	?	21	2	0	southern Arctic and south	Milliron 1949, Burks 1979c, Janšta et al. 2017
Mymaridae	15	93	369	276	all ecozones	Burks 1979i, Huber and Fidalgo 1998, Huber and Lin 2000, Huber 2004, 2012, Triapitsyn et al. 2007, Triapitsyn 2017
Ormyridae	5	9	11	2	south of Taiga Cordillera and Arctic	Burks 1979b, Hanson 1992
Perilampidae	11	20	25	5	south of Taiga ecozones	Burks 1979b, Darling 1983, 1999, Darling and Miller 1991
Pteromalidae	110	293	697	404	all ecozones	Burks 1979b, Heydon 1989, 1995, Heydon and LaBerge 1988, Darling 1991, Bouček 1993, Gibson and Vikberg 1998, Gibson 2000, 2003, 2009, Gibson and Floate 2001,
Signiphoridae	4	1	7	6	Mixedwood Plains	Gordh 1979, Woolley 1988
Tetracampidae	2	4	4	0	Montane Cordillera, Boreal Shield, Mixedwood Plains, Atlantic Maritime	Yoshimoto 1978, Bouček 1993
Torymidae	32	58	148	90	southern Arctic and south	Grissell 1976, 1979, 1995, 2000, Janšta et al. 2017
Trichogrammatidae	6	34	113	79	southern Arctic and south	Burks 1979j, Pinto 1999, 2004
**Total Chalcidoidea**	**500**	**1210**	**3301**	**2135**		
**Superfamily Mymarommatoidea^16^**						
Mymarommatidae ^16^	0	2	1	0	Boreal Shield, Mixedwood Plains, Atlantic Maritime	Gibson et al. 2007, Huber et al. 2008
**Superfamily Ceraphronoidea**						
Ceraphronidae	35	26	275	249	southern Arctic and south	Dessart 1975, Muesebeck 1979, Dessart and Cancemi 1987, Johnson and Musetti 2004
Megaspilidae	35	21	101	80	southern Arctic and south	Muesebeck 1979, Dessart 1981, 1987, Dessart and Cancemi 1987, Johnson and Musetti 2004
**Total Ceraphronoidea**	**70**	**47**	**376**	**329**		
**Superfamily Cynipoidea**						
Cynipidae ^17^	110	62	133	71	southern Arctic and south	Burks 1979a, Shorthouse and Ritchie 1984, Ritchie 1993, Melika and Abrahamson 2002, Ronquist et al. 2015
Figitidae ^18^	36	60	620	560	all ecozones	Burks 1979a, Ferrer-Suay et al. 2012, 2014, Ritchie 1993, Ros-Farré and Pujade-Villar 2009, 2011, 2013
Ibaliidae	4	4	2	0	all except Arctic	Burks 1979a, Liu and Nordlander 1992, 1994, Ritchie 1993
Liopteridae	0	1	0	0	Mixedwood Plains	Burks 1979a, Ritchie 1993, Liu et al. 2007
**Total Cynipoidea**	**150**	**127**	**755**	**631**		
**Superfamily Evanioidea**						
Aulacidae	17	18	6	0	southern Arctic and south	Townes 1950, Carlson 1979a, Smith 2001, Deans et al. 2018
Evaniidae	4	4	2	0	Mixedwood Plains	Townes 1949b, Carlson 1979a, Deans 2005, Deans et al. 2018
Gasteruptiidae	10	8	8	0	all except Arctic	Townes 1950, Deans et al. 2018
**Total Evanioidea**	**31**	**30**	**16**	**0**		
**Superfamily Stephanoidea**						
Stephanidae ^19^	2	2	0	0	Pacific Maritime, Western Interior Basin, Mixedwood Plains	Townes 1949a, Carlson 1979d, van Achterberg 2002, Aguiar 2004
**Superfamily Trigonaloidea**						
Trigonalidae ^20^	4	4	4	0	Pacific Maritime, Boreal Shield, Mixedwood Plains, Atlantic Maritime	Townes 1956, Carlson 1979b, Carmean 1995, Carmean and Kimsey 1998
** Aculeata **						
**Superfamily Chrysidoidea^21^**						
Bethylidae	35	27	83	56	all except Arctic	Evans 1978, Krombein 1979a,
Chrysididae ^22^	33	84	101	17	all except Arctic	Krombein 1979a, Bohart and Kimsey 1982, Kimsey and Bohart 1991
Dryinidae	35	50	150	100	all except Arctic	Krombein 1979a, Olmi 1984
Embolemidae	2	2	0	0	south of Taiga ecozones	Krombein 1979a, Olmi 1995
**Total Chrysidoidea^21^**	**105**	**163**	**334**	**173**		
**Superfamily Apoidea**						
**Apoidea: Apiformes**						Hurd 1979, Sheffield et al. 2017
Andrenidae	250	182	153	20–40*	southern Arctic and south	Bouseman and LaBerge 1979, LaBerge 1969, 1973, 1977, 1980, 1986, 1987, 1989, LaBerge and Bouseman 1970, LaBerge and Ribble 1972, 1975, Donovan 1977, Ribble 1968, 1974
Apidae ^23^	189	206	213	50*	all ecozones	Cockerell 1903, LaBerge 1956a, b, 1961, Mitchell 1962, Daly 1973, Rightmyer 2008, Williams et al. 2014
Colletidae	45	54	50	5–10*	all ecozones	Stephen 1954, Mitchell 1960, Snelling 1966a, b, 1970
Halictidae	110	200	184	15*	all ecozones	Mitchell 1960, Roberts 1972, 1973, McGinley 1986, Gibbs 2010, Dumesh and Sheffield 2012, Gibbs et al. 2013, Heron and Sheffield 2015
Megachilidae	150	210	209	20*	All ecozones	Sandhouse 1939; Michener 1938a, b, c, 1939, 1947; Timberlake 1943; Mitchell 1962; Rightmyer et al. 2010; Sheffield et al. 2011; Gonzalez and Griswold 2013
Melittidae	2	3	3	0	Montane Cordillera, Prairies, Mixedwood Plains, Atlantic Maritime	Michez and Patiny 2005, Michez and Eardley 2007, Payette 2013, Sheffield and Heron 2018
**Total Apiformes**	**746**	**855**	**812**	110–135*		
**Apoidea: Spheciformes**						Bohart and Menke 1976, Krombein 1979d, Pulawski 2018, Sann et al. 2018
Ammoplanidae ^24^	?	5	4	0	Western Interior Basin, Prairies, Mixedwood Plains	Smith 2008, 2009
Ampulicidae ^24^	?	2	0	0	Boreal Shield, Mixedwood Plains	Kohl 1893, Rohwer 1917, Bradley 1934, Buck 2004
Astatidae ^24^	?	19	12	0	southern Arctic and south	Parker 1962, 1969, 1972, Steiner 1973, Finnamore 1982, 1997, Buck 2004, Ratzlaff 2016, Sheffield 2017
Bembicidae ^24^	?	81	56	0	southern Arctic and south	Parker 1917, Bradley 1920, Bohart and Horning 1971, Finnamore 1982
Crabronidae s. str.^24^	?	181	127	0	southern Arctic and south	Finnamore 1982, 1997, Bohart 1976, Leclercq 2000, 2006, 2008, 2012
Mellinidae ^24^	?	2	2	0	Prairies, Mixedwood Plains	Strickland 1947, Finnamore 1982, Buck 2004
Pemphredonidae ^24^	?	53	58	0	southern Arctic and south	Strickland 1947, Finnamore 1982, Buck 2004
Philanthidae ^24^	?	56	26	0	southern Arctic and south	Scullen 1965, 1968, Bohart 1966, Bohart and Grissell 1975
Psenidae ^24^	?	34	27	7	southern Arctic and south	Finnamore 1980, 1982, 1983, Buck 2004, Ratzlaff 2016
Sphecidae	225	64	50	0	southern Arctic and south	Menke 1965
**Total Spheciformes**	**225**	**497**	**362**	**7**		
**Total Apoidea**	**971**	**1352**	**1174**	117–142*		
**Vespoidea s. lat.^25^**						
**Superfamily Formicoidea**						
Formicidae	139	212	302	90	southern Arctic and south	Francoeur 1997, Glasier and Acorn 2014, Glasier et al. 2013, 2016, Canadian Endangered Species Conservation Council 2016, AntWeb 2018, Bolton 2018; J Heron pers. comm.
**Superfamily Pompiloidea^26^**						
Mutillidae ^27^	30	26	13	0	south of Boreal Cordillera and Taiga ecozones except Pacific Maritime	Krombein 1979b, Pitts 2007, Williams et al. 2012, Brothers and Lelej 2017
Pompilidae	150	107	107	0	southern Arctic and south	Evans 1950, 1951a, b, Townes 1957, Krombein 1979c, Wasbauer and Kimsey 1985, Finnamore 1997, Paiero et al. 2010
Sapygidae	6	7	9	2	all except Arctic	Krombein 1979b, Kurzenko 1996
**Superfamily Scolioidea^28^**						
Scoliidae	2	4	2	0	Western Interior Basin, Mixedwood Plains	Krombein 1979b, MacKay 1987
**Superfamily Tiphioidea^26^**						
Sierolomorphidae	1	2	4	2	Pacific Maritime, Boreal Cordillera, Boreal Plains, Prairies Mixedwood Plains	Evans 1961, Krombein 1979b, Finnamore 1997, Buck et al. 2005, Lelej and Mokrousov 2015
Tiphiidae ^29^	25	31	13	0	south of Boreal Cordillera and Taiga ecozones	Allen 1966, 1968, 1971, Krombein 1979b, Kimsey and Wasbauer 2006
**Superfamily Thynnoidea^26^**						
Chyphotidae ^30^	?	1	0	4*	Western Interior Basin, Prairies	Mickel 1967, Krombein 1979b
Thynnidae ^29^	?	3	3	2*	south of Taiga ecozones except Pacific Maritime	Pate 1947, Krombein 1979b, Kimsey 2009
**Superfamily Vespoidea s. str.**						
Rhopalosomatidae	2	1	0	2*	Mixedwood Plains	Townes 1977a, Lohrmann et al. 2012
Vespidae	100	96	102	6	southern Arctic and south	MacLachlan 1980, Buck et al. 2008, Buck et al. 2012, Kimsey and Carpenter 2012, Canadian Endangered Species Conservation Council 2016, R Longair pers. comm.
**Total Vespoidea s. lat.^25^**	**455**	**490**	**555**	**108**		
**Total Aculeata**	**1531**	**2005**	**2063**	**398–423**		
**Total Hymenoptera**	**6028**	**8757**	**18,454**	**10,366–10,391**		

**^1^**Classification mostly follows Goulet and Huber (1993), except Cynipoidea follows Ronquist (1999), Vespoidea s. lat. follows Pilgrim et al. (2008), except for Myrmosidae which is now considered part of Mutillidae (Brothers and Lelej 2017), Diaprioidea follows Sharkey (2007), Platygastroidea follows Sharkey et al. (2012), Chalcidoidea follows Heraty et al. (2013) and Janšta et al. (2017) and “Spheciformes” follows Sann et al. (2018). **^2^**Barcode Index Number, as defined in Ratnasingham and Hebert (2013). **^3^**Undescribed/unrecorded species numbers calculated using number of BINs minus recorded species except for families marked with an asterisk (*) for which additional taxonomic and/or faunal information was available to modify estimate. ^3A^See figure 1 in Langor (2019) for a map of ecozones. **^4^**References listed for taxa above the family level are major works such as catalogues, distributional checklists, revisions and online taxonomic resources that cover multiple families within the higher taxon (usually all families). References in family rows are generally species-level revisions, checklists or catalogues of entire families, subfamilies, tribes or genera including most or all species recorded in Canada at the time of their publication. **^5^**Orussoidea (Orussidae) part of Siricoidea in Masner et al. (1979). **^6^**Pamphilioidea called Megalodontoidea in Masner et al. (1979) and including Xyeloidea (Xyelidae). **^7^**Diaprioidea of current study equivalent to Diapriidae of Masner et al. (1979). **^8^**Diapriidae of Masner et al. (1979) also included Diapriidae of current study. **^9^**Platygastroidea of current study equivalent to Scelionidae + Platygastridae of Masner et al. (1979). **^10^**Platygastridae of current study includes Scelionidae totals of Masner et al. (1979) (synonymized by Sharkey 2007). **^11^**Proctotrupoidea of Masner et al. (1979) included Platygastroidea and Diaprioidea of current study, but omitted Pelecinidae. **^12^**Azotidae previously part of Aphelinidae (Heraty et al. 2013), but not known from Canada in 1979 (Gordh 1979). **^13^**Eulophidae of current study includes Elasmidae totals of Masner et al. (1979) (synonymized by Gauthier et al. 2000). **^14^**Eurytomidae mistakenly omitted from Masner et al. (1979). About 80 species were recorded from Canada at the time. **^15^**Megastigmidae previously part of Torymidae (Janšta et al. 2017). Number of species recorded in Torymidae by Masner et al. (1979) uncertain. **^16^**Mymarommatidae (= Mymarommidae) included in Chalcidoidea by Masner et al. (1979). **^17^**Unclear what taxa were included in Cynipidae by Masner et al. (1979), but likely part of current Figitidae. **^18^**Eucoilidae and Alloxystidae recognized by Masner et al. (1979), but included in Figitidae in totals of current study. **^19^**Stephanidae included in Ichneumonoidea in Masner et al. (1979). **^20^**Trigonalidae included in Chrysidoidea (= Bethyloidea) in Masner et al. (1979). **^21^**Chrysidoidea referred to as Bethyloidea in Masner et al. (1979). **^22^**Chrysididae of current volume includes Chrysididae and Cleptidae totals of Masner et al. (1979). **^23^**Apidae of current volume includes Apidae, Anthophoridae and Xylocopidae of Masner et al. (1979). **^24^**All families in “Spheciformes” included in Sphecidae in Masner et al. (1979) (uncertain how many of each family were recorded). **^25^**Vespoidea s. lat. value for Masner et al. (1979) equal to sum of their Scolioidea, Formicoidea and Vespoidea. Species totals for superfamilies in Vespoidea s. lat. not calculated because of major differences in composition between current study and Masner et al. (1979). **^26^**Superfamily not recognized in Masner et al. (1979). See text for discussion of how classification differs from Pilgrim et al. (2008). **^27^**Mutillidae total from Masner et al. (1979) may include some species that are now placed in Chyphotidae. Also includes Myrmosidae of Pilgrim et al. (2008), now considered part of Mutillidae (Brothers and Lelej 2017). See text for how these taxa are related. **^28^**Scolioidea of Masner et al. (1979) includes Mutillidae, Rhopalosomatidae, Sapygidae, Scoliidae, Sierolomorphidae and Tiphiidae. **^29^**Tiphiidae total from Masner et al. (1979) may include some species that are now placed in Thynnidae. See text for how these taxa are related. **^30^**Masner et al. (1979) likely included Chyphotidae in Mutillidae totals.

The survey of Masner et al. (1979) gave estimates on the number of undescribed or unrecorded species in Canada, with the caveat “Such estimates are especially difficult (because so many families of Hymenoptera are inadequately known)…” The same caveat applies to the current survey; however, the use of Barcode Index Numbers (BINs) (Ratnasingham and Hebert 2013) based on 2% or greater sequence divergence of DNA barcodes in the Barcode of Life Data System (BOLD) provides us with new tools to help estimate hitherto unrecorded species diversity for some groups. For the purposes of this assessment, rather than add a somewhat subjective value for each family, the numbers of unrecorded species for most families were calculated based on the number of known BINs minus the number of described recorded species. This method is used for all families except those for which ongoing revisionary studies or faunal surveys have indicated that the number of BINs is not a good estimate of the total number of unrecorded species in Canada (these values noted in Table 1 with an asterisk). The BIN totals are current as of July 2018, but are likely underestimated for most superfamilies (see discussion at end of Faunal Analysis section).

The distribution of Hymenoptera families across ecozones in Canada (Rankin et al. 2011) is incompletely known. The species data in the checklists on which Table 1 is based were sorted by political unit, not ecozone, and it was not possible to go back and determine precise ecozones based on specimen locality data for all taxa (although this was done for the smaller families). In some cases, such as for the families of sawflies, knowledge of host plant distributions helped us make judgements on the ecozone ranges. For most families, there is no supporting information such as host distributions, habitats, or climatic ranges that can help discern whether range gaps are due to lack of sampling or whether a species is actually absent from an ecozone. Therefore, subjective decisions were made concerning whether to extrapolate the known range to encompass areas where there are sampling gaps. Most large Hymenoptera families span all the southern ecozones of Canada and some of them also range into the Arctic.

The information sources from which the data in Table 1 were taken is not exhaustive but instead contains the most important sources. Additional sources are noted for many taxa in the main body of text. In addition to the literature, specimens of all families in the Canadian National Collection of Insects, Arachnids and Nematodes (CNC), Ottawa were also examined, providing a rich source of data to aid completion of Table 1. Family and superfamily level classification mainly follows Goulet and Huber (1993), except as noted in the footnotes of Table 1.

## Overview of Hymenoptera diversity

The current study reports 8757 described species compared to approximately 6000 in 1979 (once omissions and overestimates in Masner et al. (1979) are taken into account). The approximately 2750 new records of Canadian Hymenoptera added since 1979 represent a 46% increase and an average of 71 new records/year. These figures indicate that Hymenoptera is one of the most diverse and relatively least known orders of insects in Canada. Masner et al. (1979) estimated 10,637 undescribed/unrecorded species which, when combined with the 6000 recorded species totalled 16,637 species for Canada. This means that in 1979, approximately 36% of the total estimated species were described/recorded. Currently, we estimate 10,366–10,391 undescribed/unrecorded species (Table 1) in an estimated total fauna of 19,148 species, of which approximately 46% are described/recorded.

### Sawflies (previously suborder Symphyta)

All sawflies are herbivorous as larvae, except for members of the superfamily Orussoidea which are parasitoids of larval wood-boring Coleoptera and Hymenoptera (Middlekauff 1983, Goulet 1993). Sawflies occupy a paraphyletic grade at the base of the phylogeny of Hymenoptera, and Xyeloidea is consistently recovered as the sister group to all other Hymenoptera (Sharkey et al. 2012, Peters et al. 2017). Global estimates for species richness range between 8000 and 8300 species (Taeger et al. 2010, Huber 2017). Species of all seven superfamilies of sawflies and 12 of the 14 extant families, except Blasticotomidae and Megalodontesidae, are recorded in Canada. Masner et al. (1979) recorded eleven families in Canada; Anaxyelidae (*Syntexislibocedrii* Rohwer) was subsequently recorded from southern British Columbia (Goulet 1992). Furthermore, Masner et al. (1979) recorded 443 described species of sawflies, whereas the current survey has 710, an increase of 60.3% (Table 1). As in most parts of the world (especially the northern hemisphere), the sawfly fauna of Canada is dominated by the family Tenthredinidae, representing 75% or more of the total species.

Masner et al. (1979) estimated only 131 undescribed/unrecorded sawfly species, which was a significant underestimate as more than twice as many species (267) have been subsequently recorded from Canada. This large increase was a result of extensive work by many authors, most notably David R. Smith, who authored the Nearctic catalogue (Smith 1979a) and many revisions (e.g., Smith 1979b, 1989), and Henri Goulet (e.g., Goulet 1986, 1996). In addition, extensive collecting and faunal surveys (e.g., Goulet 1987) have contributed to our knowledge. A catalogue of world species (Taeger et al. 2010) and an accompanying searchable, electronic taxonomic database ECatSym (Blank et al. 2012) is also a rich source of information about all sawflies, including the Canadian fauna.

Examining DNA barcode data alone, it may appear that most of the diversity of Canadian sawflies has now been discovered as the number of BINs is lower than recorded species in all families except Siricidae, Cimbicidae, and Pergidae, all of which have fewer than 30 described species. This is likely misleading as surveys of Tenthredinidae in northern Europe, which has been surveyed much more intensively than northern North America, reveal a much greater diversity than northern Canada (e.g., Prous et al. 2017), suggesting that the Canadian fauna includes many more species than currently known. Ongoing revisions indicate that at least 200 undescribed species of Tenthredinidae are present in Canada, mostly in the subfamilies Nematinae and Tenthredininae (H Goulet pers. comm.) which emphasizes the fact that more collecting and DNA barcoding of Canadian sawflies is required.

### Apocrita

Ichneumonoidea

In terms of described species, Ichneumonoidea is the largest superfamily of Hymenoptera, both in Canada (4202 species) (Table 1) and the world (47,177 species) (Yu et al. 2016). It is also the largest superfamily of insects in Canada comprising an impressive 10.8% of the 38,925 described insect species recorded (Langor 2019). There are two families, Ichneumonidae and Braconidae. Almost all ichneumonoids are parasitoids of other insects (Wahl and Sharkey 1993), the exceptions being a few genera that are parasitoids of spiders or prey on arachnid eggs (Townes 1969) and a few that are known to be phytophagous (e.g., Marsh 1991). The electronic catalogue of Ichneumonoidea (Yu et al. 2016) is an invaluable resource for accessing knowledge of the superfamily, including taxonomy, nomenclature, distribution, biology, references, etc.

Braconidae is the second largest family of Hymenoptera in Canada (1165 described species recorded; Table 1) and the world (21,221 described species; Yu et al. 2016). The current total is a 40.4% increase over the 830 species reported by Masner et al. (1979). Masner et al. (1979) estimated that there were 3200 undescribed/unrecorded braconid species in Canada. The number of BINs of Braconidae recorded in Canada in BOLD is 3411 (2246 more BINs than the number of described species recorded in Canada). Therefore, even though the estimate of undescribed Canadian braconid species by Masner et al. (1979) may appear to be a slight overestimate, studies on the percentage of undescribed microgastrine braconids in Canada and elsewhere in the world (e.g., Rodriguez et al. 2013) indicate that the number of undescribed braconids in Masner et al. (1979) may be accurate or even conservative. Good progress has been made on Canadian Braconidae since Masner et al. (1979) including a catalogue of all Nearctic species (Marsh 1979a, b) and keys to all New World genera (Wharton et al. 1997).

There are 25,285 described species of Ichneumonidae worldwide (Yu et al. 2016) and the actual fauna is estimated to be greater than 100,000 species (Gauld et al. 2002). Based on described, recorded species, Ichneumonidae is the most speciose family in Canada with 3037 species (Table 1) which represents approximately 35% of all described species of Hymenoptera recorded in Canada and 7.8% of all described species of insects (Langor 2019). The number of described, recorded species of ichneumonids reported in Masner et al. (1979) was 2001, including one species of “Pachylommatidae”, now called Hybrizontinae, that is considered part of Ichneumonidae (Sharkey and Wahl 1992). Since 1979, the number of described species of ichneumonids recorded in Canada has increased by 1036 (51.8%). Most of this increase was because of major revisions of Nearctic taxa (e.g., Dasch 1979, 1984, 1988, 1992, Townes 1983, Townes et al. 1992) as well as increased distributional knowledge via faunal surveys (e.g., Schwarzfeld 2014).

Masner et al. (1979) estimated that there were 5000 undescribed or unrecorded species of ichneumonids in Canada, but no discussion was provided to justify the estimate. There are 4748 known BINs for Canadian Ichneumonidae, ~1700 more BINs than recorded species (Table 1). Comparing the number of BINs to the estimated total number of species from Masner et al. (1979) (2001 known + 5000 anticipated = 7001), the current study has a shortfall of 2253 species. Whether an additional 2253 species of Canadian Ichneumonidae await discovery is unclear, but certainly, considering the very high diversity of Ichneumonidae in northern latitudes (e.g., 97 morphospecies recorded from Ellesmere Island, Nunavut; Timms et al. 2013), it is likely that many more species of Ichneumonidae remain to be collected and DNA barcoded in the less well-sampled regions of Canada (especially the North).

Diaprioidea

Historically, Diaprioidea was grouped within Proctotrupoidea (e.g., Masner et al. 1979, Muesebeck 1979); however, Sharkey (2007) found that Proctotrupoidea was polyphyletic and therefore removed Diapriidae and related families to a new superfamily. Diaprioidea includes four families (Sharkey 2007, Sharkey et al. 2012) of which Diapriidae is the most speciose, with 2048 species reported by Huber (2017), the other three families accounting for an additional 61 species. Two families are known in Canada, Diapriidae and Diapriidae (Table 1). Masner et al. (1979) considered Diapriidae part of Diapriidae, but the phylogenetic analysis of Sharkey et al. (2012) raised the subfamily Ismarinae to family status within Diaprioidea. Almost all Diaprioidea, for which the biology is known, are parasitoids of Diptera, although Diapriidae are hyperparasitoids of Dryinidae (Hymenoptera) parasitizing leafhoppers (Hemiptera: Cicadellidae) (Masner 1993a).

Masner et al. (1979) recorded 150 described species of Diapriidae (including Diapriidae) in Canada. The current study records 177 described species of Diapriidae and eight Diapriidae, which together is a 23.3% increase from 1979. The ratio of BINs to described species is 4.1 (763 BINs) implying that many undescribed/unrecorded species of diaprioids occur in Canada. Despite the relatively poor state of knowledge of Diaprioidea in Canada, there are some valuable resources on the group including keys to the New World genera of Diapriinae (Masner and García 2002) and a world catalogue with species distributions by biogeographical region (Johnson 1992). All information on Diapriidae has been updated and placed by N Johnson and colleagues on Hymenoptera Online (various contributors 2018), including additional distributional information and relevant literature. Masner (1976a) revised the world species of Diapriidae.

Platygastroidea

Masner et al. (1979) and Muesebeck (1979) classified Platygastridae and Scelionidae within Proctotrupoidea, but later classifications (e.g., Masner and Huggert 1989) separated these two families from Proctotrupoidea to form Platygastroidea. Sharkey (2007) synonymized the two on the basis of paraphyly of Scelionidae with respect to Platygastridae. Almost all known Platygastroidea are egg parasitoids of a variety of insect orders as well as of spiders (Masner 1993b). Huber (2017) indicated that there are 5385 known species worldwide.

Masner et al. (1979) recorded 150 described species of Scelionidae and 100 Platygastridae in Canada. The current study records only 160 described species combined, which means that the numbers reported by Masner et al. (1979) included undescribed species. Masner et al. (1979) estimated that there may be up to 300 unrecorded species of Platygastroidea in Canada, i.e., more unrecorded species than recorded. The ratio of BINs to recorded species in the current study supports the fact that Platygastroidea are very poorly known in Canada and, in fact, this value (14.3) is the highest of any Hymenoptera superfamily in Canada (2287 BINs). Based on this, there may be more than 2,100 undescribed/unrecorded species of Platygastroidea in Canada, making it the third largest superfamily of Hymenoptera in the country (after Ichneumonoidea and Chalcidoidea), and easily the most poorly known. Despite the apparent dearth of knowledge of the group, there have been many studies of Platygastroidea since 1979, such as a world revision of Platygastridae s. str. (Townes and Townes 1981), the world catalogue of Johnson (1992) that included species in the former Scelionidae (but not Platygastridae s. str.), keys to world genera of Scelioninae (Masner 1976b) and many revisions of Nearctic genera (e.g., Masner 1983a, b, Ritchie and Masner 1983). All information on Platygastroidea is available on an extensive website devoted to the systematics of the superfamily (Johnson 2018) and much of this knowledge has also been uploaded into Hymenoptera Online (various contributors 2018).

Proctotrupoidea

Proctotrupoidea is comprised of eight families (Huber 2017), of which five are present in Canada (Table 1). Huber (2017) recorded 448 described species worldwide. Masner et al. (1979) placed Pelecinidae within its own superfamily but it is now classified within Proctotrupoidea (Johnson and Musetti 1999). Conversely, Masner et al. (1979) included Platygastridae, Scelionidae, and Diapriidae in Proctotrupoidea but the first two families now comprise Platygastroidea (Masner 1993b) and the latter is placed in Diaprioidea (Sharkey 2007).

All proctotrupoids are parasitoids. Species of Proctotrupidae have been reared from Coleoptera and Diptera (Masner 1993a). Pelecinidae parasitizes Scarabaeidae (Coleoptera) (Johnson and Musetti 1999), Heloridae has been reared from Chrysopidae (Neuroptera) (Townes 1977b), Roproniidae from a sawfly (Masner 1993a), and Vanhorniidae from Eucnemidae (Coleoptera) (Deyrup 1985).

There are 73 described species of Proctotrupoidea in Canada, compared to 66 reported in Masner et al. (1979) (Table 1). Proctotrupidae is the largest family with 67 species and the other four families have one or two species each. The ratio of BINs to described species for the superfamily is 0.92. The world catalogue by Johnson (1992) summarizes the species and regional distributions, and additional references on the superfamily can be found on Hymenoptera Online (various contributors 2018).

Chalcidoidea and Mymarommatoidea

Chalcidoidea is comprised of 23 extant families (Heraty et al. 2013, Janšta et al. 2017), of which 18 are present in Canada (Table 1). Masner et al. (1979) included Mymarommatidae (as Mymarommidae) as a family within Chalcidoidea, but Gibson (1986) removed Mymarommatidae from Chalcidoidea, and Noyes and Valentine (1989) were the first to treat the taxon as a superfamily. Chalcidoidea have been reared as parasitoids from a wide variety of insect orders as well as some Arachnida and the nematode family Anguinidae, but a few are predators (using more than one host to complete development) and some are phytophagous (Gibson 1993). The biology of Mymarommatoidea is unknown although one has been reared from a bracket fungus and most are collected in shady, moist areas such as deciduous forests (Gibson et al. 2007, Huber et al. 2008).

Chalcidoidea is one of the world’s most diverse superfamilies of organisms. More than 22,700 species are described (Huber 2017), but Heraty et al. (2013) estimated that there may be as many as 500,000 species worldwide. Masner et al. (1979) recorded 16 families of Chalcidoidea in Canada (not including Mymarommatidae) but neglected to include Eurytomidae in their treatment. Other differences include Elasmidae (*Elasmus* Westwood), now classified within Eulophidae (Gauthier et al. 2000), Azotidae (*Ablerus* Howard), now classified in its own family instead of within Aphelinidae (Heraty et al. 2013), and Megastigminae removed from Torymidae and classified as Megastigmidae (Janšta et al. 2017). Masner et al. (1979) recorded 500 described species of Chalcidoidea in Canada, whereas the current survey records 1210 (a 142% increase which is the largest percentage increase of new species records over that time period for any Hymenoptera superfamily in Canada). The great increase in the number of recorded Chalcidoidea is a reflection of the large amount of work that has been done on this group (see Table 1). Chapters in the Nearctic catalogue were provided by Burks 1979b-j, Gordh 1979, and Grissell 1979, including distributional ranges in Canada. A key to the genera of Nearctic Chalcidoidea was published by Gibson et al. (1997). These publications have facilitated biological and faunal distributional studies for many taxa. All of the literature and taxonomic information to date is freely available in an online catalog, Universal Chalcidoidea Database (Noyes 2017). Despite the great amount of progress made on Canadian Chalcidoidea since 1979, the number of BINs (3301) is 2.7 times the number of recorded species and, based on this number, it is estimated that an additional 2135 undescribed/unrecorded species occur in the country (Table 1). The most speciose families in Canada based on BINs are Eulophidae (1373), Pteromalidae (697), and Mymaridae (369).

Mymarommatoids are very small wasps with a body length less than 1 mm (Gibson 1993). Huber (2017) reported ten described species worldwide. In Canada, they are only recorded in the east (Gibson et al. 2007), but they are also known in Montana (Hatten et al. 2010); therefore their range likely spans Canada from west to east. Masner et al. (1979) recorded no described species of Mymarommatidae in Canada, but predicted one unrecorded species to be present. The current survey records two species in Canada (Table 1) and Huber et al. (2008) provided keys to the described Nearctic species. There is one BIN for Mymarommatoidea from Canada in BOLD.

Ceraphronoidea

Ceraphronoidea is comprised of two families, Ceraphronidae and Megaspilidae, and there are 603 described species worldwide (Huber 2017). All ceraphronoids are parasitoids, most usually of Diptera, or hyperparasitoids of Hymenoptera, but they have also been associated with Hemiptera, Thysanoptera, Lepidoptera, Neuroptera and Mecoptera (Masner 1993c). There are 47 recorded Canadian species of Ceraphronoidea (Table 1), but this is one of the most poorly studied groups of Hymenoptera which is reflected in a BIN to recorded species ratio of 8.0 (10.6 for Ceraphronidae and 4.8 for Megaspilidae). This implies that there are over 375 species of Ceraphronoidea in Canada, of which most (329) remain to be described/recorded. Masner et al. (1979) recorded 70 described species in Canada (35 for each family), but this number included undescribed species based on their knowledge of the literature and examination of specimens in the CNC. Muesebeck (1979) provided a catalogue for the Nearctic species with Canadian distributions and Johnson and Musetti (2004) published a world catalog with distributions by region. Dessart and Cancemi (1987) provided keys to genera.

Cynipoidea

Cynipoidea (gall wasps and allies) is another understudied group of Hymenoptera. There are approximately 3200 species described globally (Huber 2017). The biology of the superfamily is diverse, with Cynipidae being mostly phytophagous gall-makers (but also inquilines in galls of other insects), whereas species in other families are parasitoids (e.g., Ibaliidae on siricid and anaxyelid sawflies; eucoiline Figitidae on cyclorrhaphous Diptera) (Ritchie 1993, Ronquist 1999). The current study records 127 described species of Cynipoidea in Canada which is slightly fewer than the total (150) reported in Masner et al. (1979). The number of BINs of Cynipoidea is 755, which, if representative of the total number of species, means that there could be as many as 631 unrecorded species in Canada (Table 1). The BIN to described species ratio is 5.9 showing that Cynipoidea is the third most poorly known superfamily in Canada, after Platygastroidea and Ceraphronoidea.

The classification of the families of Cynipoidea was previously contentious, but appears to have been stabilized with recognition of five families worldwide (Ronquist 1999) of which four are recorded in Canada: Cynipidae, Figitidae (including the former Charipidae, Eucoilidae and Alloxystidae), Ibaliidae and Liopteridae (Ritchie 1993, Ronquist 1999). Liopteridae was not recorded from Canada in Masner et al. (1979), but one specimen (now lost) was collected near Hamilton, Ontario (Liu et al. 2007). There have been a few revisions and reviews since 1979 for Cynipidae (e.g., Melika and Abrahamson 2002, Ronquist et al. 2015). Figitidae is the largest family and has the most gaps in knowledge. Some subfamilies are well-studied, e.g., Aspiceratinae (Ros-Farré and Pujade-Villar 2009, 2011, 2013). A world catalogue is available for Charipinae (Ferrer-Suay et al. 2012) as are keys to Nearctic genera and a species checklist (Menke and Evenhuis 1991). In contrast, other subfamilies are lacking in revisions and literature, especially the diverse Eucoilinae. Up to date nomenclature and literature has been added to Hymenoptera Online (various contributors 2018) but the last Nearctic catalogue with distributional data for the entire superfamily was Burks (1979a).

Evanioidea

Evanioidea (ensign wasps and allies) is a small superfamily with 1130 species globally (Huber 2017) that, for Canada at least, appears to be relatively well-known. This is mainly because the group is mostly tropical and only a few genera and species have ranges that extend to northern latitudes. There are three families, all of which are present in Canada: Aulacidae, Evaniidae (ensign wasps), and Gasteruptiidae. Aulacidae are parasitoids of wood-boring Coleoptera and sawflies, Evaniidae lay their eggs in the oothecae of cockroaches, and Gasteruptiidae have been reared from nests of solitary bees or wasps where they prey on one or more eggs or larvae (Mason 1993). There are 30 described species of Evanioidea in Canada, compared to 31 reported by Masner et al. 1979 (Table 1). The ratio of BINs to recorded species is only 0.53 (16 BINs vs 30 recorded species) which shows that more DNA barcode sampling is required. This is most evident for Evaniidae for which only two Canadian DNA barcodes are present in BOLD, despite four recorded species. Few or no unrecorded Canadian species of Evanioidea are expected. In terms of literature, keys to the Nearctic species are available, for Aulacidae (Townes 1950), Gasteruptiidae (Townes 1950, Smith 1996) and Evaniidae (Townes 1949). Carlson (1979a) provided the Nearctic catalogue for Evanioidea, Deans (2005) updated the nomenclature for Evaniidae, and Smith (2001)published a world catalogue of Aulacidae. Up to date information about Evanioidea is available at Evanioidea Online (Deans et al. 2018).

Stephanoidea

Stephanoidea is a small, mostly tropical group of Hymenoptera comprised of one family, Stephanidae; 342 species are known globally (Huber 2017). They are long, slender insects (body length up to 2 cm) that parasitize wood-boring Coleoptera (Mason 1993). There are two species in Canada (Table 1), the same number reported by Masner et al. (1979). One species is in the west and one in the east. There are keys to the Nearctic species (Townes 1949a) and no additional species are expected in Canada. An updated key to world genera is provided by van Achterberg (2002) and Aguiar (2004) published a world catalog including distributions by country. A summary of literature on the family is found online (Aguiar 2005). Stephanidae was included in Ichneumonoidea by Masner et al. (1979) but this classification is no longer commonly accepted (Aguiar 2005).

Trigonaloidea

Trigonaloidea, comprised of one family, Trigonalidae, lay eggs on leaves which are eaten by caterpillars or sawfly larvae. Except for some extralimital species which are primary parasitoids of pergid sawflies (Raff 1934), eggs of most trigonalid larvae do not continue development following ingestion unless the host is parasitized by an ichneumonoid wasp or tachinid fly or is captured by a vespid wasp (Carmean 1995). Globally there are 92 known species (Huber 2017). There are four species of Trigonalidae recorded in Canada (Table 1), the same number reported by Masner et al. (1979) and also four BINs from Canadian specimens in BOLD. Townes (1956) provided keys to the four Nearctic species. It is unlikely that additional species will be recorded from Canada.

### Aculeata

Aculeata is a demonstrably monophyletic group (Branstetter et al. 2017) comprised of the superfamilies Chrysidoidea, Apoidea, and the assemblage of families that previously comprised the Vespoidea (hereafter called Vespoidea s. lat.). Aculeata includes many of the most recognizable groups of Hymenoptera, including the bees, ants, and vespid wasps. There are 2005 described species of Aculeata recorded in Canada which represents 22.9% of all recorded described Hymenoptera species (Table 1). Except for the Chrysidoidea, the group is relatively well-known based on the ratios of BINs to recorded species.

Chrysidoidea

Chrysidoidea includes 6780 species worldwide (Huber 2017) classified into seven families (Gauld and Hanson 1995), of which four are present in Canada (Table 1). They are parasitoids (or occasionally kleptoparasites) of a wide range of insect orders including Coleoptera, Lepidoptera, Hymenoptera, Phasmatodea, Embioptera (for the extralimital Sclerogibbidae), and Hemiptera (Finnamore and Brothers 1993, Gauld and Hanson 1995). Based on molecular data, they are hypothesized to be the sister group to the rest of Aculeata (Heraty et al. 2011, Peters et al. 2017) or a paraphyletic grade of two clusters of families at the base of Aculeata (Branstetter et al. 2017).

There are 163 described species of Chrysidoidea recorded in Canada, compared to 105 reported by Masner et al. (1979), a 55.2% increase. All Nearctic families have been revised since 1979. For Chrysididae (cuckoo wasps), a Nearctic revision was published (Bohart and Kimsey 1982) as well as a world review with species checklists (Kimsey and Bohart 1991). Olmi (1984) published a world revision of Dryinidae, with a supplement (Olmi 1991). The other major family in Canada, Bethylidae, was revised for the Nearctic by Evans (1978), and it appears that the number of described species in Canada reported by Masner et al. (1979) (35) was a slight overestimate of the number of Canadian species currently known (27 based on Evans (1978) and material in the CNC). Finally, Olmi (1995) revised the small family Embolemidae, but his revision did not change the number of species recorded in Canada (two). In terms of undiscovered diversity in Canada, the proportion of BINs to described species is 2.04 for the superfamily, indicating that there may be as many as 173 undescribed/unrecorded species of Chrysidoidea present in Canada, most of which belong to Bethylidae and Dryinidae.

Apoidea

Just under 30,000 described species of Apoidea are known globally (Huber 2017), with approximately two thirds representing the bees (Michener 2007). In total, the number of Apoidea species recorded for Canada has increased by approximately 39% since 1979 (1352 vs 971). Within this superfamily, the Spheciformes grade (Sphecidae sensu Masner et al. 1979) is now regarded as multiple families (Sann et al. 2018). The Crabronidae s. lat. was until recently the largest of the families, with many more than 400 species in Canada. However, the recent splitting of Crabronidae (Sann et al. 2018) resulted in several subfamilies being raised to family level as follows: Astatidae, Bembicidae, Crabronidae s. str. (previously Crabroninae), Mellinidae, Pemphredonidae, Philanthidae, and Psenidae. In addition, the subtribe Ammoplanina (previously in Pemphredoninae) was also raised to family status. Collectively, these eight families are represented by 431 species in 68 genera (Table 1). In addition to the families in the former Crabronidae, the eleven genera of Sphecidae s. str. are represented by 64 species, with a BIN to recorded species ratio of 0.78. Finally, there are two species of Ampulicidae (in two genera) from Canada but neither have been barcoded yet and no other species are expected in Canada (the only other two Nearctic species known are both from the southern United States (Krombein 1979d).

Classification of bees (Apiformes) has also changed since Masner et al. (1979), specifically with the merging of the non-corbiculate apid families Anthophoridae and carpenter bees (i.e., Xylocopidae sensu Masner et al. 1979) with the corbiculate apids (i.e., bumble bees and honey bee) into the single family Apidae (Michener 2000, 2007), resulting in six families of bees in Canada: Andrenidae, Apidae, Colletidae, Halictidae, Megachilidae, and Melittidae. Sheffield et al. (2017) recently summarized the bees of Canada, indicating that there were 855 species, though the number is likely higher when unique BINs without accompanying species-level identification are considered, especially for the poorly studied taxa *Sphecodes* Latreille (Halictidae), *Nomada* Scopoli (Apidae), and *Osmia* Panzer (Megachilidae).

Both Spheciformes and Apiformes are relatively well known; for the former, a global catalogue of species and distributional information, based on published literature, is well-maintained (Pulawski 2018). This resource, in addition to works published since 1979 (e.g., Finnamore 1983, 1997, Buck 2004, Ratzlaff 2016), has increased our knowledge of sphecid wasps (Sphecidae s. lat.), and was used to provide the summaries in Table 1. For Apiformes, Sheffield et al. (2017) provided a recent summary of Canadian species, including information on DNA barcodes, and an online catalogue for species is also available (Sheffield 2018). For bees, many revisions have occurred since Masner et al. (1979), specifically for the Canadian fauna (e.g., Gibbs 2010, Sheffield et al. 2011, Dumesh and Sheffield 2012, Onuferko 2017), or those that have included Canada in their coverage (e.g., Gibbs 2011, Rehan and Sheffield 2011, Gibbs et al. 2013).

Vespoidea s. lat.

Vespoidea s. lat. is comprised of all Aculeata that do not belong to Chrysidoidea or Apoidea (i.e., all superfamilies listed below), and is globally represented by more than 29,000 species (Huber 2017). Historically, the monophyly of the group has been equivocal. The catalogue of Hymenoptera of America North of Mexico (Krombein et al. 1979) divided the group into separate superfamilies, as did the survey of Masner et al. (1979), although these two studies differed slightly in the composition of several superfamilies. Later, morphology-based, cladistic analyses either refuted Vespoidea’s monophyly (e.g., Rasnitsyn 1988), or supported it (Brothers and Carpenter 1993). With the introduction of molecular data and a re-evaluation of the way in which characters were divided into states and polarized in earlier morphological studies (e.g., Brothers and Carpenter 1993), a consensus appears to have been reached that Vespoidea is not monophyletic, and alternative classifications have been suggested (e.g., Pilgrim et al. 2008). More recent molecular phylogenetic analyses (Branstetter et al. 2017, Peters et al. 2017) have also refuted the monophyly of Vespoidea but ambiguity still exists about the relationships of the taxa and how they relate to Apoidea, in particular because of differences in taxon choice between analyses and differences in topology correlated with differing phylogenetic methods. Because of this, the classification used here follows the suggested arrangement of Pilgrim et al. (2008) with the exception of Myrmosidae which is considered a subfamily of Mutillidae (Brothers and Lelej 2017). There are 490 described species of Vespoidea s. lat. recorded in Canada.

Formicoidea

Formicidae (the ants) was placed by itself in all molecular studies noted above, either as the sister group of Apoidea (Branstetter et al. 2017, Peters et al. 2017) or, in the preferred topology of Pilgrim et al. (2008), as sister group to Apoidea + Scolioidea. Formicidae is one of the great radiations of Hymenoptera with more than 16,000 described species (AntWeb 2018), but they are relatively poorly represented in Canada with only 212 described species recorded (Canadian Endangered Species Conservation Council 2016, J Heron pers. comm.), compared to 139 reported by Masner et al. (1979), a 52.5% increase (Table 1). In addition to the 2016 report on the conservation status of all Canadian species by province and territory, several regional checklists are available (Francoeur 1997 for the Yukon, Glasier and Acorn 2014 for the grasslands, Glasier et al. 2016 for Saskatchewan) as well as keys to workers of Alberta (Glasier et al. 2013). Our study records 302 BINs for ants (ratio to described species = 1.42), therefore there are likely ca. 90 additional species yet to be recorded in Canada. Considering the relatively good knowledge of ant taxonomy and distributional ranges, especially in northern latitudes, this is somewhat surprising, but it illustrates that even for supposedly well-known groups, our knowledge of the Canadian fauna is not complete. The ant taxonomic community is one of the most well-organized in entomology, with many resources including an online taxonomic and bibliographic catalogue (Bolton 2018) and an online database of specimen records, images and biological information (AntWeb 2018).

Pompiloidea

This group includes the velvet ants (Mutillidae), spider wasps (Pompilidae), and sapygid wasps (Sapygidae). Pompiloidea was not recognized by Masner et al. (1979). Instead, Pompilidae was placed with Vespidae in their Vespoidea s. str., and the other taxa were included in Scolioidea.

All pompiloids are parasitoids: Pompilidae on spiders (Day 1988), or in one case, a Phalangiidae (Opiliones) (Evans 1948); Mutillidae on other Aculeata, but also less commonly on Diptera, Lepidoptera, Coleoptera and Blattodea (Brothers and Finnamore 1993); and Sapygidae on bees and vespid wasps (Krombein 1979b). Some Pompilidae are kleptoparasitoids of other pompilids (Townes 1957).

The majority of Canadian diversity in this superfamily is in Pompilidae, with 107 of the 140 species (Table 1). Masner et al. (1979) listed 150 described species from Canada, but this appears to have been a slight overestimate. Pompilidae is a relatively poorly studied group in North America with only a few Nearctic faunal surveys since 1979 (e.g., Wasbauer and Kimsey 1985 for California, Finnamore 1997 for the Yukon, Sugar et al. 1999 for oak savannahs in southern Ontario). Nearctic identifications rely on the revisions of Evans (1950, 1951a, b) and Townes (1957). The most current Nearctic catalogue is Krombein (1979c), but many taxon names in this work are no longer valid and must be updated with reference to more recent, non-Nearctic catalogues (e.g., Wahis 1986, 2006). The ratio of BINs to recorded species is 1.0; however, as it is the second largest family in the Vespoidea s. lat. and it is relatively poorly studied, there could certainly be undescribed/unrecorded species.

Masner et al. (1979) recorded 30 described species of Mutillidae (including Chyphotidae) in Canada. The current study records 26 mutillids and one chyphotid (the latter now considered part of Thynnoidea). The number of recorded mutillids includes those of the subfamily Myrmosinae. This group was considered its own family by Pilgrim et al. (2008), but was moved back into Mutillidae by Brothers and Lelej (2017). There are only 13 BINs of Mutillidae from Canada on BOLD, and more sampling of this family is required. Mutillidae is a relatively well-studied family in North America with recent revisions of several major taxa (e.g., Pitts 2007, Williams et al. 2012); therefore, there is a good taxonomic foundation for surveying the Canadian fauna. Finally, Masner et al. (1979) recorded six species of Sapygidae and the current study has seven (Table 1), but there are nine BINs, therefore barcoded voucher specimens at Guelph need to be examined to determine which undescribed/unrecorded species may be present in Canada. Sapygidae is a relatively poorly studied family. Krombein (1979b) catalogued the Nearctic species including five Canadian species and Kurzenko (1996) provided a key to the Nearctic genera.

Scolioidea

Scolioidea, as defined by Pilgrim et al. (2008), consists of only one family, Scoliidae, in Canada (Krombein 1979b). Scolioidea of Masner et al. (1979) included six families (see footnote 28 in Table 1). Four species of Scoliidae are recorded from Canada, an increase from two species in Masner et al. (1979) (Table 1). All scoliids are parasitoids of Coleoptera, mostly Scarabaeoidea, but rarely Curculionoidea (Brothers and Finnamore 1993). Only two BINs have so far been recorded for Scoliidae from Canada, therefore more sampling is required. Historically, the classification of the family has been unstable, but there is now some consensus following publication of a world checklist (Osten 2005). There are 560 known species globally (Huber 2017). Very few studies on the Nearctic fauna have been done since the catalogue of Krombein (1979b), although MacKay (1987) treats the species of the southwestern US and has a key that includes all four species recorded in Canada.

Tiphioidea

Pilgrim et al. (2008) found that the family Tiphiidae was polyphyletic. The subfamilies Tiphiinae and Brachycistidinae clustered together, and therefore, these taxa were placed in Tiphiidae s. str. Their study related Tiphiidae s. str. to the monotypic family Sierolomorphidae, placing both families within Tiphioidea. Tiphioidea was not recognized by Masner et al. (1979). See Thynnoidea (below), for discussion of the placement of the other subfamilies previously belonging to Tiphiidae.

Tiphiidae are ectoparasitoids of Coleoptera (Brothers and Finnamore 1993). The current study records 31 species of Tiphiidae in Canada (Table 1). This compares to 25 species reported by Masner et al. (1979), a total which likely included one or more species that are now classified in Thynnidae. The ratio of BINs to recorded species is only 0.42, suggesting a need for more collecting and DNA barcoding. There have been no major revisions of Nearctic Tiphiinae since HW Allen’s efforts in the 1960s and 1970s (e.g., Allen 1966, 1971). Kimsey and Wasbauer (2006) provided a taxonomic checklist of the Brachycistidinae of the western Hemisphere.

Two species of Sierolomorphidae are currently recorded from Canada, up from one species reported by Masner et al. (1979); however, BOLD has four BINs from Canada for this family suggesting that undescribed/unrecorded species exist. Evans (1961) provided keys for the six Nearctic species. The hosts are unknown.

Thynnoidea

Phylogenetically, the five other subfamilies of Tiphiidae s. lat. (Kimsey 1991) clustered together in Pilgrim et al. (2008) and the valid name for this group is Thynnidae. Furthermore, Pilgrim et al. (2008) found that Thynnidae was the sister group of two subfamilies of Bradynobaenidae (Chyphotinae and Typhoctinae) which together, were raised to family status with the valid name Chyphotidae. Thynnoidea was not recognized by Masner et al. (1979).

In Canada, only three species of Thynnidae are recorded (Table 1), although a further two species are known (C Sheffield unpubl. data). Most Thynnidae are parasitoids of beetles (e.g., Methocinae on Cicindelinae), although one species of the extralimital subfamily Diamminae has been reared from mole crickets (Orthoptera: Gryllotalpidae) (Brothers and Finnamore 1993). Only three BINs are currently recorded for Thynnidae in Canada. Pate (1947) provided keys to the Nearctic genera.

One species of the family Chyphotidae is known from Canada (Mickel 1967) (Table 1). Little is known of the biology of Chyphotidae, but a species of the extralimital genus *Typhoctes* Ashmead has been found on immature Solifugae (Arachnidae) (Brothers and Finnamore 1993). Likely the Canadian species was included in Masner et al. (1979) as one of the species of Mutillidae recorded from Canada. There are no BINs for Chyphotidae from Canada. There are four species known from states bordering southwestern Canada (Mickel 1967), therefore more species are likely to occur in Canada.

Vespoidea s. str.

The analyses of Pilgrim et al. (2008) and Branstetter et al. (2017) found that Vespidae and Rhopalosomatidae are sister groups. In contrast, Vespoidea s. str. of Masner et al. (1979) was comprised of Vespidae and Pompilidae.

There are 96 species of Vespidae (yellow jackets, potter wasps, hornets, paper wasps, and allies) known from Canada (Canadian Endangered Species Conservation Council 2016, R Longair pers. comm.). The current number is slightly fewer than the number cited in the 2016 report (101) because the current list excludes several adventive species that are not considered to be established. Masner et al. (1979) recorded 100 species and the number of BINs is 102. The relative similarity of these totals illustrates the good level of knowledge that exists for Canadian Vespidae, especially for the northeastern Nearctic (Buck et al. 2008, 2012).

Rhopalosomatidae is a small family with only four genera worldwide (Brothers and Finnamore 1993). It has previously been proposed as the sister group of Pompilidae (Brothers 1999), related to Formicidae, Scoliidae, and Vespidae (Brothers and Carpenter 1993) or related to Mutillidae, Sapygidae, Scoliidae, Sierolomorphidae, and Tiphiidae (Masner et al. 1979). Very little is known of the biology of the family, and the only known hosts are crickets (Orthoptera: Gryllidae) (Townes 1977a). Only one species of Rhopalosomatidae is recorded from Canada, the brachypterous *Olixonbanksii* (Brues) from southern Ontario (Lohrmann et al. 2012). Masner et al. (1979) listed two species from Canada, which we assume included *Rhopalosomanearcticum* Brues, but we have not seen Canadian specimens of this species. It is recorded from Kentucky and Maryland, so its range could extend into Canada. A third genus, *Liosphex* Townes, is also recently recorded from Kentucky (*L.boreus* Lohrmann) (Lohrmann and Ohl 2010), therefore this genus may also be discovered in Canada in the future. There are no Canadian BINs for Rhopalosomatidae.

## Faunal analysis

The results of the current survey have re-confirmed that Hymenoptera is one of the major constituents of biodiversity in Canada with 8,757 described species recorded (Table 1). The percentage of the Nearctic Hymenoptera fauna that is present in Canada cannot be determined precisely because Nearctic species totals have not been updated for some families since Krombein et al. (1979). However, total described species numbers for North America north of Mexico are known for two of the largest superfamilies, Ichneumonoidea and Chalcidoidea, which together comprise approximately two thirds of all described species recorded in Canada. Approximately 55% of Nearctic ichneumonoids are recorded in Canada (4202 of 7647) and approximately 34% of the chalcidoids (1210 of 3567). Together, 48.3% (5412 of 11,214) of these two superfamilies are recorded in Canada. If similar percentages exist for the remaining one third of species, then it can be estimated that roughly half of the described species of Hymenoptera in the Nearctic north of Mexico are recorded in Canada. At a global level, Canada has approximately 5.7% of the 153,410 described species of Hymenoptera in the world as tabulated by Huber (2017).

In terms of composition of the Hymenoptera of Canada, just over three quarters of the described, recorded species (77.3%) belong to three superfamilies: Ichneumonoidea (4202 species: 48.0%), Apoidea (1352 species: 15.4%) and Chalcidoidea (1210 species: 13.8%). The sawfly superfamily Tenthredinoidea is the fourth largest with 595 species (6.8% of total species) and Vespoidea s. lat. is fifth (490 species: 5.6%). The overall composition of Hymenoptera in Canada differs slightly if one considers total species (recorded species plus our estimates of unrecorded species). There are as many as 19,148 species with the following proportions: Ichneumonoidea (42.6%), Chalcidoidea (17.5%), Platygastroidea (12%), Apoidea (7.8%), and Tenthredinoidea (4.2%).

Canada’s Hymenoptera faunal structure is similar to other countries in northern latitudes. For example, Broad (2014) found the following proportions for described species recorded from Britain and Ireland: total species (7764), Ichneumonoidea (3913 species: 50.4 %), Chalcidoidea (1717 species: 22.1%), Tenthredinoidea (492 species: 6.4%), Apoidea (385 species: 4.9%) and Platygastroidea (362 species: 4.7%). The higher percentage of Apoidea recorded in Canada relative to Britain and Ireland is probably a reflection of greater diversity of habitats in Canada, especially hot, dry regions such as the Western Interior Basin, Prairies, and Mixedwood Plains ecozones which have a high diversity of Apoidea relative to cooler, more northern areas (Buck 2004, Sheffield et al. 2014). Relative to the whole world, Canada has a much higher percentage of described species of Ichneumonoidea (48.0% in Canada vs 30.8% for the whole world), slightly fewer Apoidea (15.4% vs 19.3%), approximately the same percentage of Chalcidoidea (13.8% vs 14.8%) and slightly more Tenthredinoidea (6.8% vs 4.7% worldwide). The higher percentage of Ichneumonoidea in northern latitudes compared to the tropics was discussed by previous authors (e.g., Janzen 1981, Gauld 1987), but more recent work on tropical ichneumonoids has demonstrated that this pattern is likely artefactual because of incomplete surveying of parasitoids in tropical areas of the world (Santos and Quicke 2011, Veijalainen et al. 2012, Timms et al. 2016). Apart from Ichneumonoidea, the other major difference between the composition of Hymenoptera in Canada compared to that of the entire world is the percentage of Vespoidea s. lat. (5.6% in Canada vs 19.0% in the entire world). Most vespoid families are predominantly tropical (Brothers and Finnamore 1993) and some, such as Chyphotidae, Rhopalosomatidae, Scoliidae, and Thynnidae have only one or a few species with ranges barely extending to southern Canada (see Table 1).

With respect to quantification of the number of introduced species of Hymenoptera in Canada, these numbers are available for some groups (e.g., sawflies, ants, bees and vespid wasps), but they are very poorly known for the parasitoid groups which encompass greater than 80% of the described species diversity of Hymenoptera in Canada. The reason for this lack of knowledge is a combination of poor distributional and taxonomic knowledge in many groups (both in Canada and elsewhere), as well as more than 100 years of well-meaning, but poorly documented, deliberate introductions of species for biological pest control that have obscured the native ranges of species in many groups. We can state that approximately 5% of sawflies appear to be introduced to Canada (H Goulet pers. comm.), and the Wild Species 2015 report (Canadian Endangered Species Conservation Council 2016) provided the following percentages: 7% of ants, just more than 2% of bees, and 5% of vespid wasps.

The 46% increase (8757 vs 6000) in recorded, described species since 1979 indicates that a great deal of work has been done in the last 39 years to document Canada’s Hymenoptera, but the high number of BINs (18,454) and estimated, unrecorded species (10,366–10,391) suggests that much more work is required as fewer than half of the total species are currently recorded. At the suprafamilial level, the following groups have had relatively few newly recorded species since 1979: Ceraphronoidea, Cynipoidea, Diaprioidea, Evanioidea, Platygastroidea, Proctotrupoidea, Stephanoidea, Trigonaloidea, bees (Apoidea: Apiformes), and Vespoidea s. lat. In contrast, other groups have had significant increases in the number of recorded species (values in parentheses are the percentage increases of recorded species in the current study compared to 1979): Chalcidoidea (142%), Apoidea: Spheciformes (121%), sawflies (60%), Chrysidoidea (55%), and Ichneumonoidea (48%). The great increase in the number of recorded species in these taxa indicates a relatively low level of taxonomic and distributional knowledge in 1979 coupled with a strong research effort since that time, especially publication of the *Catalog of Hymenoptera of America North of Mexico* (Krombein et al. 1979) and many revisionary studies in these groups (see references in Table 1).

Despite the great amount of research that has been performed on many groups of Canadian Hymenoptera, some groups require much more investigation, as indicated by high ratios of total estimated species (unrecorded plus recorded species) to recorded species: Platygastroidea (14.3), Ceraphronoidea (8.0), Cynipoidea (5.9), Diaprioidea (4.1), Chalcidoidea (2.7), Chrysidoidea (2.0), and Ichneumonoidea (1.9). All other suprafamilial taxa have ratios of approximately 1.2 or less, implying that they are relatively well documented in Canada. However, the ratio of total estimated species to recorded species is not the sole indicator of taxa in most need of taxonomic and survey work. Species richness must also be considered. For example, the ratio of total estimated species to recorded species for Ichneumonidae is 1.56 which is not even in the top ten ranking for families. However, in terms of the absolute number of unrecorded species estimated in this study, Ichneumonidae (1705 unrecorded species) ranks third behind only Braconidae (2246) and Platygastridae (2127).

Although we rely heavily on BIN data to estimate the number of undocumented species in most families, we realize that this approach may not provide good estimates of species richness in Canada for all families because of incomplete DNA barcoding libraries for some families and/or inability of DNA barcodes to distinguish all species correctly. Given the relatively short length of time that DNA barcoding has been in widespread use (Hebert et al. 2003), it is not surprising that some groups, especially those that are speciose in understudied regions, e.g., Ichneumonidae in the high Arctic (Timms et al. 2013), are not completely sampled and therefore are incompletely represented in the DNA barcode library. Also, there are taxa for which the DNA barcoding region of cytochrome oxidase I does not correctly distinguish all species. For example, 50–60% of 90 species of northwestern European sawflies of the genus *Pristiphora* Latreille could not be distinguished using DNA barcodes (Prous et al. 2017, and also see the general discussion on DNA barcoding of sawflies by Schmidt et al. 2017). Within the bees, *Ceratina* Latreille, *Lasioglossum* Curtis, and *Bombus* Latreille contain some problematic taxa in which multiple species share a single BIN (Shefﬁeld et al. 2017), but these instances are rare and barcodes still permit identification to a sibling species pair or species group. In general, a large majority of hymenopteran species were able to be discriminated by barcoding in prior studies (e.g., 97.3% of European bees; Schmidt et al. 2015). In addition, hymenopteran specimens are notoriously difficult to barcode, exhibiting only a 65% recovery rate, roughly 30% lower than some orders like Lepidoptera and Diptera (Hebert et al. 2016). This poor barcode recovery is likely the product of their high adenine-thymine (AT) content (that complicates sequencing) and the demonstrated difficulties in PCR primer binding, both associated with the high rates of mitochondrial molecular evolution in Hymenoptera (Kaltenpoth et al. 2012). This low recovery rate compounds the challenge of comprehensively sampling the DNA of Hymenoptera across Canada, and thus underscores that estimation of the unknown Canadian fauna cannot rely on BINs alone. In summary, the percentage of the fauna that is documented (46%) may be under- or over-estimated; however, the actual percentage does not matter nearly as much as the stark fact that an enormous amount of work is required to document thousands of species that are hitherto unknown.
